# Hypermethylation of the Promoter Region of miR-23 Enhances the Metastasis and Proliferation of Multiple Myeloma Cells *via* the Aberrant Expression of uPA

**DOI:** 10.3389/fonc.2022.835299

**Published:** 2022-05-30

**Authors:** Qijie Ran, Dehong Xu, Qi Wang, Dongsheng Wang

**Affiliations:** ^1^ Department of Hematology, General Hospital of Central Theater Command, Wuhan, China; ^2^ Department of Neurosurgery, The Fifth People’s Hospital of Dalian, Dalian, China; ^3^ Department of Neurosurgery, The Second Affiliated Hospital of Dalian Medical University, Dalian City, China

**Keywords:** multiple myeloma, uPA, miR-23, hypermethylation, patient-derived cell lines

## Abstract

Multiple myeloma has a long course, with no obvious symptoms in the early stages. However, advanced stages are characterized by injury to the bone system and represent a severe threat to human health. The results of the present work indicate that the hypermethylation of miR-23 promoter mediates the aberrant expression of uPA/PLAU (urokinase plasminogen activator, uPA) in multiple myeloma cells. miR-23, a microRNA that potentially targets uPA’s 3’UTR, was predicted by the online tool miRDB. The endogenous expressions of uPA and miR-23 are related to disease severity in human patients, and the expression of miR-23 is negatively related to uPA expression. The hypermethylation of the promoter region of miR-23 is a promising mechanism to explain the low level of miR-23 or aberrant uPA expression associated with disease severity. Overexpression of miR-23 inhibited the expression of uPA by targeting the 3’UTR of uPA, not only in MM cell lines, but also in patient-derived cell lines. Overexpression of miR-23 also inhibited *in vitro* and *in vivo* invasion of MM cells in a nude mouse model. The results therefore extend our knowledge about uPA in MM and may assist in the development of more effective therapeutic strategies for MM treatment.

## Introduction

Multiple myeloma (MM) is a clonal malignant plasma cell disease that accumulates in bone marrow, leading to bone destruction and marrow failure as well as out-bone injury for the whole body at advanced stages (1–3). Increasing data have been indicated that the MM is often accompanied by the multiple osteolytic lesions, hypercalcemia, or kidney damage (1–5). Although therapeutic approaches to MM have been rapidly evolving, and MM is typically sensitive to a variety of cytotoxic chemotherapies, the prognosis of patients with distal metastasis (the extra-bone MM, at an advanced stage of the disease) is still poor (4–6). Bone destruction is among the most debilitating manifestations of MM and results from the interaction between myeloma cells and the bone marrow microenvironment (7, 8). During the malignant transformation of MM, the aggregation and clustering of MM cells increases, which not only breaks through the bone marrow and enters the bone but also eventually forms masses and solid tumor tissues (9–11). This feature is not only closely related to the progression of MM disease but also affects patient prognosis (9–11). Therefore, it is of value to explore the molecular metastasis of MM as it induces bone injury or distal metastasis.

The interactions between cancerous cells, extracellular matrix (ECM) or tumor microenvironment are of importance to the invasion and metastasis of human malignancies (12–14). Increasing evidence confirms that a key step in the metastasis and invasion of malignant tumor cells is the dissolution and destruction of the basal membrane, which is mainly constructed by the ECM (15–17). After this membrane is destroyed by cancerous cells, the cells can metastasize from the original site to other sites or the organs (15–17). The uPA, a serine protease involved in ECM degradation, degrades the ECM by cleaving the precursor proteins of MMPs to activate them (18–21). There are many reports on the functions and regulatory mechanisms of uPA in HCC and other solid tumor tissues (21) but not in hematological malignancies/neoplasms such as MM, a blood cancer of monoclonal plasma cells. There are only a few reports on the influence of uPA in other cells of the bone marrow/bone tissue (such as osteoclasts) on the microenvironment of bone tissue and MM cells (22–26). At the same time, the interaction between MM cells and tumor microenvironment is of great important (22–26). Therefore, it is important to explore the function and regulation mechanisms of uPA in regulating MM cells.

Here, we report for the first time and clarify the expression of uPA in MM tissues. In MM cells, miR-23 downregulates the expression level of uPA by acting on its 3’UTR. The hypermethylation of the promoter region of miR-23 leads to low expression of miR-23 and high expression of uPA. In MM cells, the overexpression of miR-23 can inhibit the tumorigenesis of MM cells in nude mice by downregulating the expression of uPA. At the same time, this study creatively uses image analysis and other quantitative methods to determine whether the morphology of the tumor tissue formed by MM cells is regular, and finally, it simulates and detects the aggregation and clustering features of MM cells.

## Materials and Methods

### Clinical Specimens, Cell Lines and Vectors

The human related materials were included MM cells and tumor tissues. The clinical specimens used in this study were (1) 35 samples of intra-marrow MM (separated from intraosseous samples); (2) 42 samples of intra-blood MM (separated from blood samples); (3) 38 samples of MM with bone-tumor tissues in the form of masses or lumps, where the MM cells form solid tumor tissue or mass in bone (bone tumor samples); and (4) 21 MM samples with long-distance metastasis, or extra-bone masses or lumps forming a solid tumor tissue or mass in other organs, developed through long distance metastasis (extra-bone tumor samples). The sample size for the four groups’ MM used has adequate power to detect a pre-specified effect size (the 1-β: 0.8; α/2: 0.025; P<0.05): the original hypothesis was that the expression level of uPA or miR-23 was not significantly different between groups; whereas the alternative hypothesis was that the expression level of uPA or miR-23 was significantly different between groups. For the bone marrow aspirate, the CD38 is used as the marker for sorting, and the CD38 positive components are retained; for the peripheral blood, the peripheral blood lymphocytes are directly separated, and then the CD38 is used as the marker for sorting, and the CD38 positive components are retained; For tumor tissue outside the bone or bone, only the tumor tissue can be collected for detection.

The enrolled patients’ inclusion and exclusion criteria: (1) Bone tumor or extra-none tumor/extraosseous (the solitary plasmacytoma), Biopsy-proven solitary lesion of bone or soft tissue consisting of clonal plasma cells; Normal random bone marrow biopsy with no evidence of clonal plasma cells; Normal skeletal survey and MRI or CT (except for the primary solitary lesion); Absence of end-organ damage, such as hypercalcaemia, renal insufficiency, anaemia, and bone lesions (CRAB) attributable to a plasma cell proliferative disorder. (2) intraosseous/intra-marrow MM (the plasma cell myeloma), Clonal bone marrow plasma cell percentage ≥ 10% or biopsy-proven plasmacytoma and ≥ 1 of the following myeloma-defining events (End-organ damage attributable to the plasma cell proliferative disorder; Hypercalcaemia: serum calcium > 0.25 mmol/L [> 1 mg/dL] higher than the upper limit of normal or > 2.75 mmol/L [> 11 mg/dL]; Renal insufficiency:creatinine clearance < 40 mL/minute or serum creatinine > 177 μmol/L [> 2 mg/dL]; Anaemia: a haemoglobin value of > 20 g/L below the lower limit of normal or a haemoglobin value < 100 g/L; Bone lesions: ≥ 1 osteolytic lesion on skeletal radiography, CT, or PET/CT≥ 1 of the following biomarkers of malignancy; Clonal bone marrow plasma cell percentage ≥ 60%; An involved-to-uninvolved serum free light chain ratio ≥100; > 1 focal lesion on MRI); (3) blood/intra-blood MM (the plasma cell leukemia), at least 20% circulating plasma cells and a total plasma cell count in peripheral blood of at least 2×10^9^/L.

Among the patient-derived cell (PDC) lines, PDC-1 and PDC-2 were directly separated, cultured, and stored. The MM clinical specimens of solid tumor tissues from PDC-3 to PDC-8 were ground in a sterile 200-mesh steel sieve with 20% FBS DMEM, and the ground cell suspension was washed with 20% FBS DMEM to obtain PDCs (27). MM cell lines used in the present work were U266 and RPMI-8226, purchased from the Cell Resource Center, Institute of Basic Medicine, Chinese Academy of Medical Sciences, Beijing, China, from the National Infrastructure of Cell Line Resources of Chinese Government. Eight PDC lines of MM cells were generated and used in the present work: (1) clonal plasma cells progressively expanded and separated within the bone marrow, PDC-1 and PDC-2; (2) cells separated from the blood/extra-bone marrow) PDC-3 and PDC-4; (3) MM cells separated from bone tumor tissues, PDC-5 and PDC-6; (4) MM cells separated from extra-bone tumor tissues, PDC-7 and PDC-8. For PDC-1–4, the cell suspension was directly cultured and frozen in liquid nitrogen tanks. For PDC-5–8, the surgically resected tumor tissue was preserved with DMEM, containing 20% FBS (27). At the same time, 20 lines of primary B cells were used as control compared with MM.

Specific preparation methods for cell lines: (1) for the MM in the bone, the bone marrow aspirate is directly sorting by CD38, and a stable MM cell line is finally obtained; (2) for the peripheral blood MM, the peripheral blood lymphocytes are directly sorting by CD38 and cultured for a long time; (3) for bone tumors, through surgical resection or bone puncture specimens, the tumor tissue was microdissected using a pre-sterilized 200-mesh steel sieve containing 20% The DMEM of FBS was ground to obtain a single-cell suspension; the single-cell suspension was washed and then cultured to obtain a patient-derived MM cell line; (4) for peripheral organ tumors, surgically resected specimens were microdissected. Then, the tumor tissue was ground with pre-sterilized 200-mesh steel sieve DMEM containing 20% FBS to obtain a single-cell suspension; the single-cell suspension was washed and then cultured to finally obtain a patient-derived MM cell line. The PDC lines were generated using the samples for one patient only. For primary B cells, healthy human peripheral blood lymphocytes (that is, peripheral blood lymphocytes obtained from the remaining part of the whole blood provided by the blood transfusion department except plasma and red blood cells) were used and primary B cells obtained by flow sorting (CD45^+^/CD19^+^).

Full-length sequences of has-pre-miR-23c (miR-23c), PLAUs with wild-type miR-23c targeting sites located in the 3’UTR, and PLAUs with mutated miR-23c targeting sites located in the 3’UTR were prepared as lentivirus particles (Vigene Coporation, Jinan City, Shandong Province, China). The MM cells were cultured and transfected with the lentivirus according to the instruction from the manufacture. MM cells were cultured and counted, and approximately 10^9^ pfu of lentivirus was inoculated per 5 x 10^6^ cells, followed by screening for stable transfection. Among them, uPA was screened by G418, and miR-23 was screened by puromycin.

### qPCR and BSP-NGS

The expression level of uPA or miR-23 in MM samples was measured using qPCR following the methods described in previous publications and instructions from the manufactures (28–30). Briefly, RNA samples of MM cells or tumor tissues were extracted using a PARISTM Kit (Thermo Fisher Scientific, Waltham, MA, USA). Next, these RNA samples were reverse transcribed with Multiscribe™ Reverse Transcriptase (Thermo Fisher Scientific) into the cDNA samples. Then the expression level of uPA and miR-23 in MM was determined using quantitative PCR (qPCR), which was performed following the methods described in previous studies (31, 32). The level of β-actin mRNA was measured as a loading control. The primers used in qPCR experiments were as follows: uPA/PLAU: forward sequence, 5’-TTGCTCACCACAACGACATT-3’; reverse sequence, 5’-ATTTTCAGCTGCTCCGGATA-3’. β-actin (the loading control) forward sequence, 5’-CACCATTGGCAATGAGCGGTTC-3’; reverse sequence, 5’-AGGTCTTTGCGGATGTCCACGT-3.’

The methylation rates of miR-23 in MM samples or cultured cells were examined by BSP-NGS following previous publications (33, 34). Briefly, the genomic DNA of clinical tissues and cell lines was extracted and isolated by using the DNeasy Blood & Tissue Kit (Cat No. 69504; QIAGEN, Hilden, Germany). For the Bisulfite (BSP) assays, the DNA samples were treated with the EpiTect Bisulfite Kit (Cat No. 59104; QIAGEN). Next, the polymerase chain reaction (PCR) assays were performed by using the Platinum II Hot-Start PCR Master Mix (Cat No. 14000012; Thermo Fisher Scientific) to amplify of the selected promoter region of miR-23 and the PCR products were directly sequenced by using Ion Torrent PGM methods (Ion PGM HI-Q View Sequencing 200 kits, Cat. No.4462921; Thermo Fisher Scientific; analytic software: Torrent Suite 5.6 and Ion Reporter 5.6, Life-technology, Thermo Fisher Scientific, Waltham, MA, USA). The The Methyl-Primer Express v1.0 software (Thermo Fisher Scientific, USA) was used to predict the CpG sites and the information of miR-23’s promoter region were: Position: hg38 chr9 (95,083,208- 95,085,207), Size: 97, and Strand: +strand. The primers used in the present work were as follows: forward sequence 5’-AGGAATTATGTGTGT TAGGAAAG-3’; reverse sequence 5’-ACAAAAATTCCCCATAAAAAA-3’. Results are given as the methylation rate (mean ± SD). During judging the results, if the miR-23s’ promoter region after BSP treatment is C, the results indicated that the CpG site was methylated; whereas if the sequence o after BSP treatment was T, the results indicated that the CpG site was not methylated. The methylation rate of miR-23’s promoter regions was calculated as the number of methylated CpG sites divided by the total number of CpG sites in the selected regions.

Taking the correlation analysis, the association between uPA expression with mir-23 expression as an example, the expression level of uPA and mir-23 can be measured in each tissue specimen. At this time, taking the expression level of uPA as the abscissa and the expression of mir-23 as the ordinate, each specimen can correspond to a data point. A group of specimens can correspond to a group of data points, which can be fitted and linearly regressed to obtain a regression equation and P value.

### Western Blotting Assays

The expression levels of PLAU in MM cell lines were measured using Western blotting assays. MM cells were transfected with the vectors (control miRNA, miR-23, miR-23 + uPA^Mut^ or miR-23 + inhibitor) and harvested for Western blotting. The expression levels of uPA in MM cells were evaluated based on the presence of antibody (Cat. No.: ab169754, Abcam, Cambridge, UK). The loading control used in the western blot section as GAPDH. The images of western blot was quantitatively analyzed by using the Image J software [National Institutes of Health (NIH), Bethesda, Maryland, USA].

### Subcutaneous Tumor Model in Nude Mice

The animal experiments were performed in a nude mice model (35). Mice 4–5 weeks old were used. MM cells were cultured in DMEM with 10% FBS and transfected with plasmids and subcutaneously injected into the mice to form tumor tissues. After 6–8 weeks of growth, the MM cells formed subcutaneous tumor tissues. The tumors were harvested and analyzed. To determine tumor volumes, tumor width and length were determined using Vernier calipers. Tumor volumes were calculated as tumor width × tumor width × length divided by 2. Tumor weights were examined using a precision-balance.

**Figure 1:**
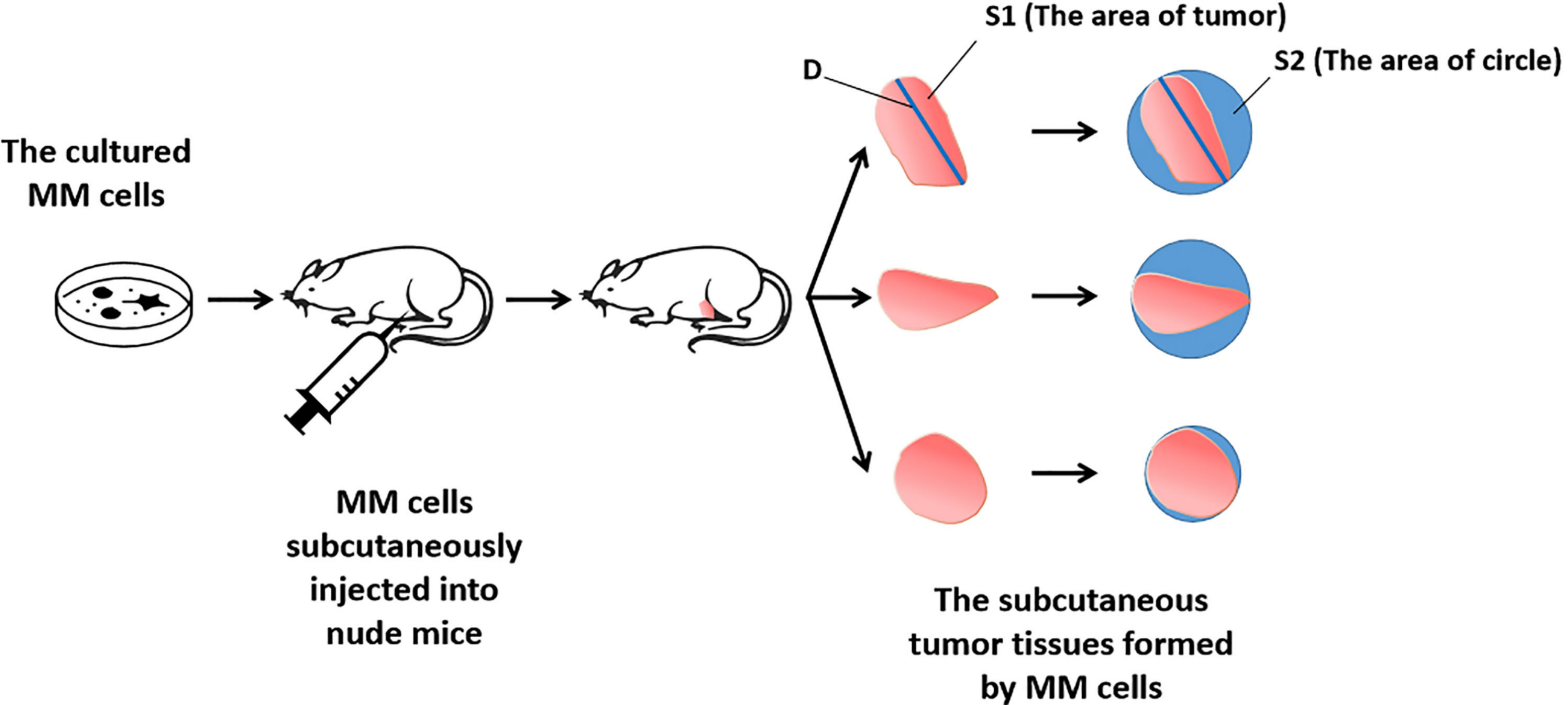
Schematic diagram of the detection method of tumor tissue morphological regularity.

**Figure 2:**
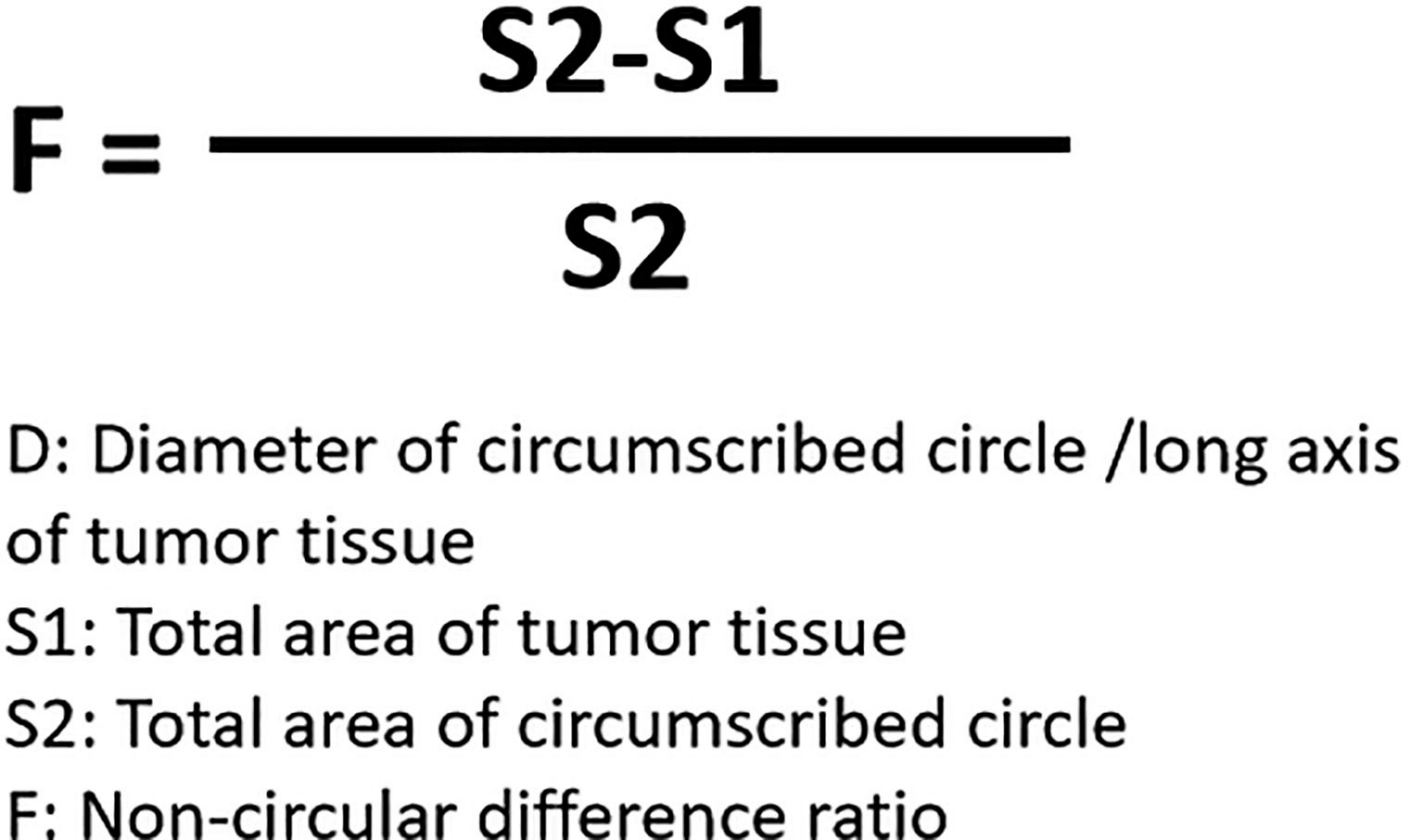
Formula for calculating the detection method of tumor tissue morphology regularity.

For the detection and discrimination of the morphological regularity of the tumor tissues, we first determined the magnitude of the long axis of each tumor tissue (tumor length). We used the image analysis software Image J to calculate the circumference of the perimeter of the tumor tissue, its diameter, and its area (36, 37). The total number of pixels in the area designated in the software is the total area of the selected area. Then the tumor tissue is subtracted out of this area, and this difference is divided by the total area selected to obtain an F value, which is evaluated to determine whether the morphology of the tumor tissue is close to that of a circle. The greater the F value, the larger the difference between the total area of the tumor tissue and the selected area, and less regular the shape of the tumor tissue. **Figure 1** is a schematic diagram of this method, and **Figure 2** shows the calculation formula. For experimental animals, each group is 8-10 animals (Each animal corresponds to a subcutaneous tumor tissue), each animal is inoculated with about 5 × 10^6^ cells, and the animal feeding cycle is 6 weeks.

### *In Vivo* Invasion of MM Cells in the Nude Mice Model

Invasion of MM cells was assessed following the methods of a previous study (21). The cells were mixed with medical hydrogels to form hydrogel drops. The drops were adhered to the surface of liver organs *via* open surgery. After 3–4 weeks of rearing, the mice were harvested, and the intrahepatic lesions/nodules formed by the MM cells were examined by pathological staining (Masson staining). Staining was quantitatively examined using the Image J software. The total depth of the liver and the invaded depth of MM cells (the depth of the intrahepatic lesions/nodules) were revealed by pixel analyses. The relative invasion of MM cells was calculated as the depth of invasion of MM cells divided by the total depth of the liver.

### Small Inhibitor of uPA Used in the Present Work

Small inhibitor of uPA, UK-371804 (Cat. No.: HY-101214), was purchased from the MedChemExpress Corporation (China Branch), Pudong New Area, Shanghai, China. UK-371804 occurs as light yellow or white powder, and was received with a purity of ≥98.0%. Organic solvents, including dimethyl sulfoxide, polyethylene glycol 400, and Tween 80 were used to resolve the pure drug powder. The UK-371804 organic solvent solution was diluted with sterilized saline. During this period, ultrasound, stirring, and vortexing were used to avoid drug precipitation (38–41). Finally, an oral liquid for oral administration to nude mice was prepared. The final concentration of UK-371804 in the oral liquid was about 1 mg/mL. Then, the anti-tumor activity of UK-371804 on MM cell tumor formation in nude mice was tested. The MM cells were injected subcutaneously to form tumor tissues. The mice were orally administered UK-371804 (1 mg/kg, 5 mg/kg, or 10 mg/kg dose) once for 3 days (38–41). The tumor volumes and weights were measured using the methods outlined above.

### Statistical Analysis

The results in the present study are given as means ± SDs from three or more biological repetitions. Significance-related analyses were examined using SPSS (Cat. No.: 9.0, IBM Corporation, Armonk, NY, USA) with the Bonferroni correction and two-way ANOVA for paired groups or the paired-sample t-test with two-ways (SPSS 16.0 statistical software; SPSS Inc., Chicago, IL, USA). The parametric linear regression the used for the co-relation analysis.

## Results

### Association Between uPA and Disease Stages/Types

**Figure 3:**
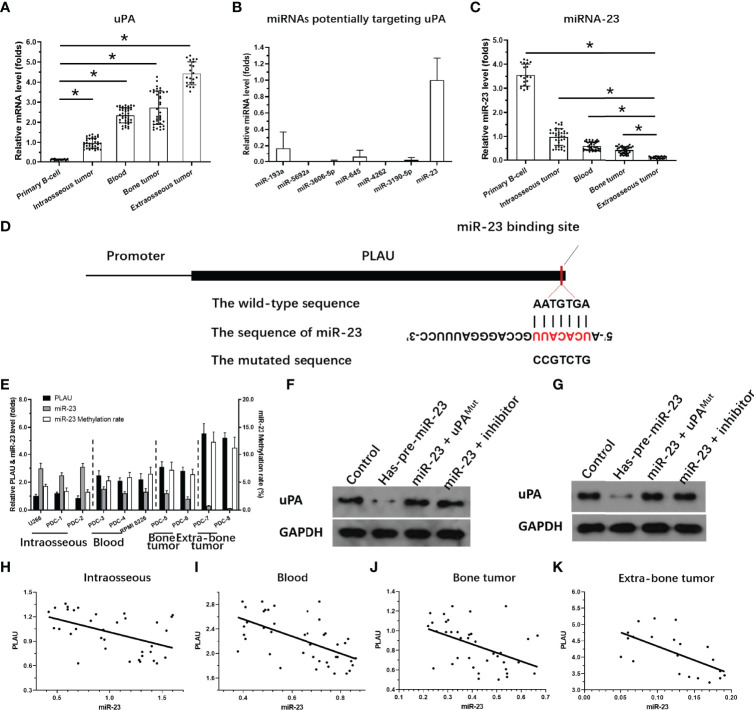
Expression of miR-23 and uPA in MM. **(A)** The expression level of uPA in MM specimensat different stages or primary B cells. **(B)** The expression level of 7 miRNA molecules (miR-23, miR-193a, miR-5692a, miR-3606-5p, miR-645, miR-4363 or miR-3190-59) potentially targeting to the 3’UTR of uPA was examined in the MM clinical specimens. **(C)** The expression level of miR-23 in MM specimens at different stages or primary B cells. **(D)** The targeting site of miR-23 at 3’UTR of uPA or mutated sequences. **(E)** The expression of uPA, miR-23, or the promoter region of miR-23 in MM cells. **(F, G)** The effects of miR-23 on uPA protein level in PDC-7 and PDC-8. **(H–K)** The association between miR-23 and uPA in MM specimens at different stages. *P<0.05.

The expression of uPA in MM clinical specimens was examined by qPCR. As shown in **Figure 3A**, expression was significantly lower in intraosseous samples than in the other three MM specimens, and highest in extra-bone tumor samples. Expression was almost the same in blood and bone tumor samples.

To clarify the possible mechanisms of the gradual increase in uPA expression in MM specimens of different degrees of malignancy, we used the online prediction tool miRDB to predict the miRNA molecules that may act on uPA. As shown in **Figure 3B**, Among the 7 miRNA molecules (miR-23, miR-193a, miR-5692a, miR-3606-5p, miR-645, miR-4363 or miR-3190-59) with the highest scores selected in the prediction results, miR-23 was clearly expressed in MM specimens, and the expression of the other 6 miRNA molecules was negative or significantly lower than that of miR-23. Therefore, we focused on the expression and mechanism of miR-23 in MM.

Next, the effects of miR-23 on uPA were assessed using assays. The results showed that miR-23, a microRNA molecule that may act on the 3’UTR of uPA, had an opposite expression trend in MM specimens from that in uPA: it was highest intraosseous specimens and lowest in extra-bone tumor samples (**Figure 3C**). The targeting sites of miR-23 in the uPA’s 3’UTR was shown as pattern diagram (**Figure 3D**). The expression level of uPA and miR-23 was also examined in MM cell lines (**Figure 3E**). Moreover, as shown in **Figures 3F** and **G**, overexpression of miR-23 in MM PDC-7 (**Figure 3F**) and PDC-8 (**Figure 3G**) led to the highest endogenous uPA level among the cell lines (**Figure 3**) and reduced the expression of uPA. The transfection of uPA with mutated miR-23 targeting sites or the antisense sequence inhibitor of miR-23 almost blocked the effects of miR-23 on the expression of uPA (**Figures 3F, G**).

The results in **Figures 3F, G** preliminarily show the effect of miR-23 on uPA expression. To further confirm the effects of miR-23 on uPA, we assessed the association between the expression levels of miR-23 and uPA in MM specimens. The results indicated that the expression level of miR-23 was negatively related with the the expression of uPA in MM specimens at different stages, and the results were shown as the scatter plot, regression equation and P values (**Figures 3H–K** and **Table 3**).

On this basis, taking primary B cells as the control, the expression levels of uPA and miR-23 in primary B cells were detected (**Figures 3A, C**). The results showed that the expression level of uPA in B cells was significantly lower than that in MM specimens (**Figure 3A**), while the expression level of miR-23 in B cells was significantly higher than that in MM specimens (**Figure 3C**). These results further confirmed the relationship between uPA and miR-23 in MM.

### Severity of Disease Stage of MM PDCs Is Associated With Aggregation and Agglomeration of Tumor Cells

**Figure 4:**
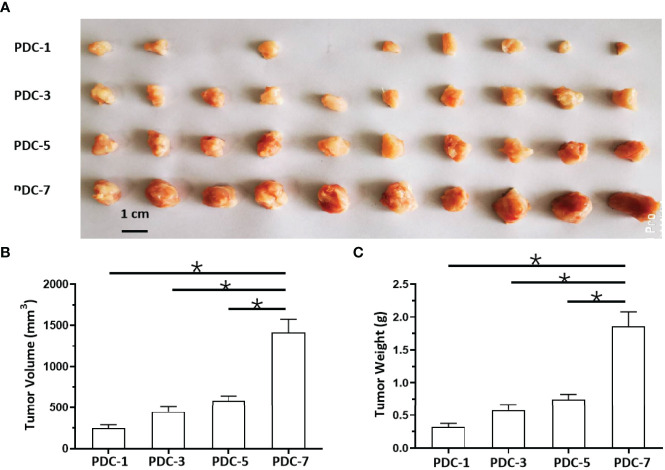
*In vivo* growth of MM cells in nude mice. PDCs (1, 3, 5, or 7) of MM were injected subcutaneously into nude mice to form tumor tissues. The results are shown as images of tumor tissues **(A)**, tumor volumes **(B)**, or tumor weights **(C)**. *P < 0.05.

Because hematological malignant cells rarely form regular solid tumors subcutaneously and because aggregation/agglomeration is often associated with the severity of MM, a subcutaneous growth model was developed for these cells (**Figure 4**). As shown in **Figure 4**, injection of a cell suspensions of MM cells (the PDC-1 [the Intraosseous MM], PDC-3 [the MM cells separated from patients’ blood], PDC-5 [the MM cells separated from the tumor tissues in bone], PDC-7 [the Extra-bone tumor]) in nude mice resulted in the formation of subcutaneous tumor tissues (the solid tumor tissues). Among the four cell lines, the tumor tissues formed by PDC-1 were uneven (Inoculated PDC-1 subcutaneously in 10 animals, and finally form tumor tissue in 7 animals) and irregularly shaped; those of PDC-3, PDC-5, and PDC-7 all formed tumor tissues that were more even and homogenous (**Figure 4A** and **Table 1**). However, those formed by PDC-7 had the greatest uniformity of traits compared with ODC-3 or PDC-5 (**Figure 4** and **Table 1**).

**Table 1:** The shape-regularity (F-values) of tumor tissues mentioned in the **Figure 4**.

Tumor No.	PDC-1	PDC-3	PDC-5	PDC-7
	F values			
1	0.232	0.174	0.113	0.023
2	0.338	0.216	0.185	0.028
3	N.A.	0.133	0.103	0.079
4	0.102	0.152	0.223	0.012
5	N.A.	0.266	0.121	0.015
6	0.244	0.166	0.176	0.016
7	0.376	0.098	0.122	0.018
8	0.285	0.122	0.121	0.125
9	0.223	0.128	0.095	0.116
10	0.196	0.186	0.106	0.177

N.A., none-application/not available.

At the same time, the MM cells of different origins and at different disease stages had different proliferation rates (**Figure 4**). Inoculated with the same number of PDCs, the volumes and weights of the tumor tissues formed by PDC-3, PDC-5, and PDC-7 were found to be significantly larger than the tumor tissues formed by PDC-1. The volume and weight of tumor tissues formed by PDC-7 were the greatest. These results indicate that the method established in this study intuitively reflects and simulates MM cells, which gradually increase their aggregation, clustering, and proliferation capabilities as the disease progresses.

### uPA Enhances Aggregation and Agglomeration

**Figure 5:**
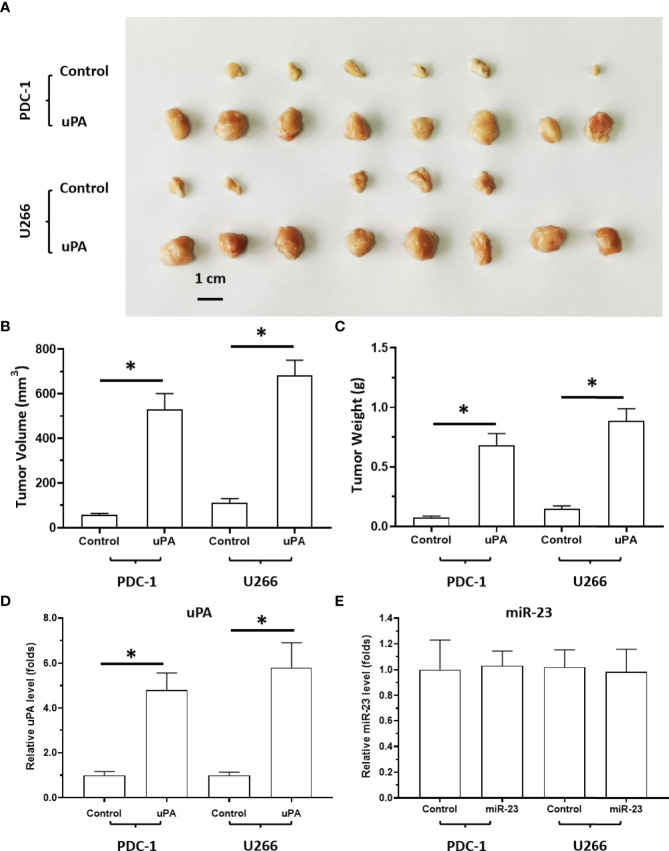
The effect of uPA overexpression on MM cells’ subcutaneous growth. The PDC-1 or U266 cells were transfected with control or uPA and injected into the subcutaneous position of nude mice to form the subcutaneous tumors. The results are shown as images of tumor tissues **(A)**, tumor volumes **(B)**, or tumor weights **(C)**. The expression level of uPA or miR-23 in the tumor tissues were examined by qPCR and shown as histogram by mean ± SD **(D, E)**. *P < 0.05.

Next, the effects of uPA on MM were examined. As shown in **Figure 5**, it was difficult for the cells (the MM cell lines U266 and PDC-1 with low levels of uPA) to form solid tumor tissues. The injection of these two cells subcutaneously into nude mice resulted in the formation of irregularly shaped and uneven tumorous tissues. The outcomes were also partial: among the H226 cells, eight animals grew five tumors; for PDC-1, eight animals grew six tumors. Overexpression of uPA in H226 or PDC-1 cells significantly upregulated the subcutaneous growth of these two cells (**Figure 5** and **Table 2**); nude mice inoculated with these two kinds of cells formed tumor tissues with significantly improved uniformity (**Figure 5** and **Table 2**). The results were shown as images of tumor tissues (**Figure 5A**), tumor volumes (**Figure 5B**) or tumor weights (**Figure 5C**). The expression level of uPA and miR-23 in the subcutaneous tumor tissues formed by transfected cells were shown as **Figures 5D** and **E**. Therefore, uPA can significantly improve the *in vivo* proliferative ability of MM cells.

**Table 2:** The shape-regularity (F-values) of tumor tissues mentioned in the **Figure 5**.

Tumor No.	PDC-1		U266	
	Control	uPA	Control	uPA
	F values			
1	N.A.	0.206	0.146	0.142
2	0.157	0.068	0.426	0.126
3	0.242	0.088	N.A.	0.089
4	0.386	0.103	0.286	0.044
5	0.256	0.062	0.313	0.046
6	0.313	0.036	0.266	0.213
7	N.A.	0.126	N.A.	0.168
8	0.042	0.077	N.A.	0.097

N.A., none-application/not available.

### Hypermethylation of miR-23 Promoter Is Associated With Low miR-23 Levels and High uPA Levels in MM

**Figure 6:**
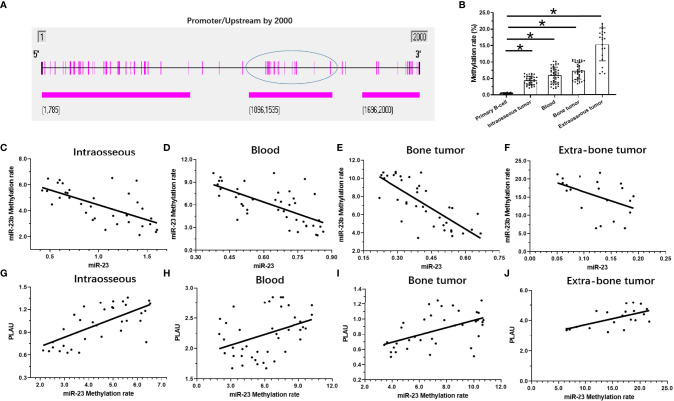
The methylation rates of miR-23’s promoter in MM. **(A)** the selected promoter region of miR-23 and island in the selected regions were shown. **(B)** the methylation rates of miR-23’s promoter region in MM clinical specimens or the primary B cells. **(C–F)** the co-relationship between miR-23 with the methylation rates of miR-23’s promoter region in different MM specimens. **(G–J)** the co-relationship between uPA/PLAU with the methylation rates of miR-23’s promoter region in different MM specimens. *P < 0.05.

The above results indicate that as the stage of MM disease (its degree of malignancy) increases, the expression level of miR-23 significantly decreases. For this reason, possible mechanisms for the loss of miR-23 expression were explored. As shown in **Figure 6**, hypermethylation of miR-23 was identified in MM specimens. In the selected (-2000 to -1) miR-23 promoter region, there are three CpG islands, and each has multiple methylation sites (**Figure 6A**). The trend for the methylation rate in the promoter region of miR-23 is exactly the opposite of that for miR-23 expression: among the four MM specimens, the methylation rate of the uPA promoter region was significantly lower in intraosseous samples than in the other three MM specimens; that of extra-bone tumor samples was the highest (**Figure 6B**). The methylation rates of uPA’s promoter region in Blood (MM with blood distribution) and MM with Bone-tumor tissues is almost the same (**Figure 6B**). To further confirm the effects of miR-23 promoter hypermethylation, we assessed the association between methylation rates and the expression levels of miR-23 and uPA in MM specimens. We found that methylation rates were negatively related to the expression of miR-23 and positively associated with the expression of uPA in MM specimens at different stages, and the results were shown as the scatter plot, regression equation and P values (**Figure 6** and **Table 3**). As an important control, the methylation rate of the miR-23 promoter region in primary B cells was also detected. As shown in **Figure 6B**, the methylation rate of the miR-23 promoter region in B cells was significantly lower than The methylation rate in MM, this result further corroborates the related results. Therefore, hypermethylation in the promoter region of miR-23 may be a mechanism for the loss of miR-23 expression in MM and the high expression of uPA.

**Table 3:** The co-relation analysis of **Figure 3** and **Figure 6**.

The association		uPA with miR-23	miR-23 with methylation	uPA with methylation
Intraosseous tumor	P values	0.0026	<0.0001	<0.0001
	Equation	Y = -0.3209*X + 1.332	Y = -2.315*X + 6.758	Y = 0.1224*X + 0.4664
Blood	P values	<0.0001	<0.0001	0.0116
	Equation	Y = -1.399*X + 3.119	Y = -10.39*X + 12.61	Y = 0.05848*X + 1.879
Bone-tumor	P values	0.0027	<0.0001	0.0008
	Equation	Y = -0.8661*X + 1.208	Y = -15.18*X + 13.51	Y = 0.04807*X + 0.5024
Extraosseous Tumor	P values	0.0026	0.0332	0.0027
	Equation	Y = -8.516*X + 5.186	Y = -49.70*X + 21.43	Y = 0.07975*X + 2.919

The “methylation” referring to “the methylation rates of miR-23’s promoter region”. *P<0.05.

### Overexpression of miR-23 Represses the Proliferation of MM Cells in a Nude Mouse Model by Targeting uPA

**Figure 7:**
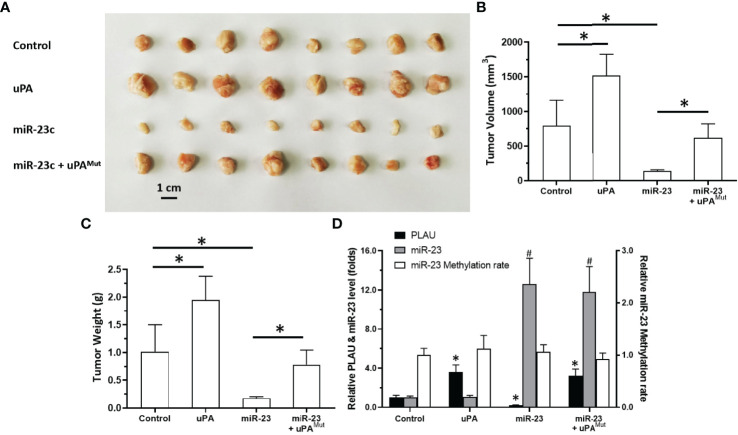
Effect of overexpression of miR-23 on uPA and in vivo proliferation in nude mice. PDC-5 of MM was transfected with vectors (control, uPA, miR-23, miR-23 + uPA^Mut^) and injected subcutaneously into nude mice. The results are shown as images of tumors **(A)**, tumor volumes **(B)**, or tumor weights **(C)**. The level of uPA expression, miR-23 expression or the methylation rates of miR-23’s promoter was shown as histogram **(D)**. ^#^P<0.05 compared with control group; *P<0.05 compared with control group.

To further elucidate the roles of miR-23/uPA in MM, each was overexpressed in MM cells. As shown in **Figure 7**, PDC-5 of MM cells formed subcutaneous tumor tissues in nude mice. Overexpression of uPA significantly promoted the subcutaneous growth of MM PDC-5. Moreover, the overexpression of miR-23 in PDC-5 not only inhibited the expression of uPA but also inhibited the subcutaneous growth of PDC-5. Co-transfection of uPA^mut^ (the uPA vector with a mutated miR-23 targeting site) almost completely blocked the inhibitory effects of miR-23 on uPA and PDC-5 (**Figures 7A–D**). Overexpression of miR-23 or uPA did not affect the methylation of miR-23 promoter in PDC-5. Therefore, miR-23 represses the proliferation of MM cells in nude mice by targeting uPA.

### Overexpression of miR-23 Represses the *In Vivo* Invasion of MM Cells in Nude Mice

**Figure 8:**
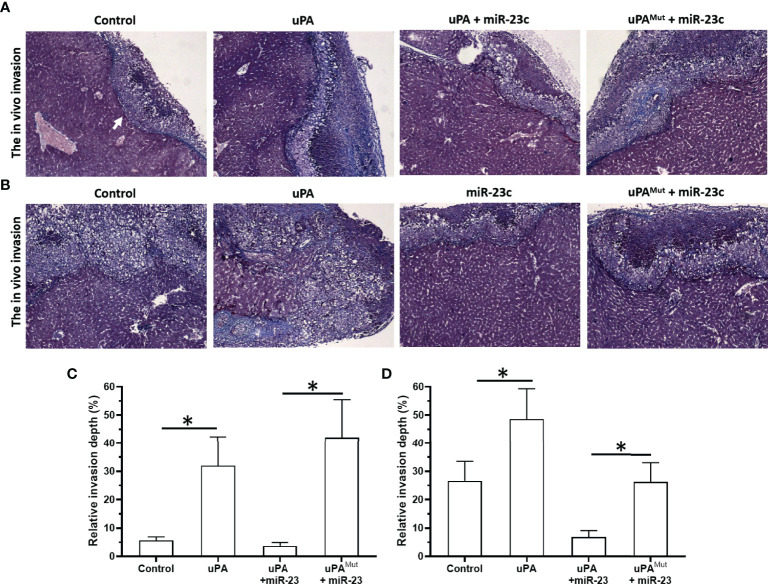
Effect of overexpression of miR-23 on uPA and *in vivo* invasion in nude mice’s liver organs. PDC-1 **(A, C)** or PDC-5 **(B, D)** of MM was transfected with vectors (control, uPA, miR-23 + uPA, miR-23 + uPA^Mut^ for PDC-1 or control, uPA, miR-23, miR-23 + uPA^Mut^ for PDC-5) mixed with hydrogel to form drops. The hydrogel drops were adhered to the surface of nude mice’s liver organs, and the intrahepatic invasion of MM cells were measured using Masson staining **(A, B)** and the quantitative results of A and B **(C, D)**. The magnification power is 400×. *P < 0.05.

Next, we tested an *in vivo* invasion model (**Figure 8**). MM cell suspension was mixed with medical gel to form droplets, and then the droplets were adhered to the surfaces of the livers of nude mice. MM cells can destroy the liver capsule and gradually invade the liver to form tumor microlesions. For PDC-1 cells, the overexpression of uPA promoted intrahepatic invasion of PDC-1. At the same time, the co-transfection of miR-23 + uPA and miR-23 inhibited the expression of uPA and inhibited the invasion induced by uPA. However, in the co-transfection of the miR-23 + uPA^Mut^ group, miR-23 did not inhibit the expression of uPA^Mut^ and the invasion induced by uPA overexpression.

Next, the effects of miR-23/uPA on MM cells were also examined in PDC-5. Although the endogenous expression of PDC-5 uPA was greater than that of PDC-1, the overexpression of uPA still significantly promoted the intrahepatic invasion of PDC-5. On this basis, because the background expression of uPA in PDC-5 is higher than that of PDC-1, miR-23+uPA was not co-transfected, but miR-23 was directly overexpressed to downregulate uPA in PDC-5. The results showed that compared to the control group, miR-23 inhibited the intrahepatic invasion of PDC-5. After this, with the overexpression of miR-23, uPAMut was co-transfected, and miR-23 no longer had an inhibitory effect on PDC-5 intrahepatic invasion. These results confirm that miR-23 inhibits the invasion of MM cells by targeting 3’UTR of uPA.

### Treatment of uPA Inhibitor Suppresses the Subcutaneous Growth of MM Cells

**Figure 9:**
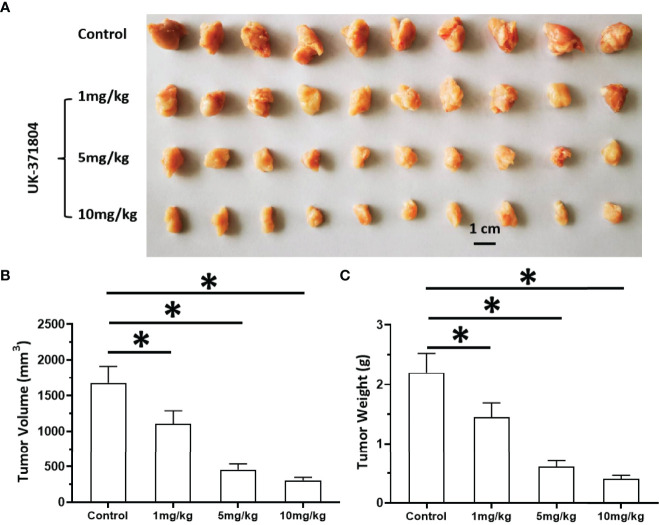
Antitumor effects of uPA small molecular inhibitor on the *in vivo* proliferation of MM cells. PDC-7 of MM was injected subcutaneously into nude mice. The mice received 1 mg/kg, 3 mg/kg, or 10 mg/kg UK-371804 *via* oral administration. The results are shown as images of tumors **(A)**, tumor volumes **(B)**, or tumor weights **(C)**. *P < 0.05.

The above results indicate that uPA is an ideal intervention target for MM treatment. To this end, we tested whether targeting uPA can inhibit the proliferation of MM cells. After inoculating MM PDC-9 cells subcutaneously into nude mice, oral gavage treatment was administered, after which tumor tissues were collected to determine tumor volume and tumor weight (**Figure 9**). The oral uPA inhibitor UK-371804 inhibited the tumorigenic effects of PDC-7 in nude mice in a dose-dependent manner. This indicates that uPA is an ideal intervention target for MM, and small-molecule inhibitors targeting uPA show clear anti-tumor activity against MM.

## Discussion

Current MM staging standards can be divided into three stages, based on the Durie and Salmon staging standards (1975), ISS international prognostic staging standards (2005), and revised R-ISS international prognostic staging standards (2015) (42–46). These staging systems are mainly based on some biochemical indicators (serum β2 microglobulin ≥ 5.5 mg/L, hypercalcemia, and so forth), bone damage (progressive osteolytic lesions, and so forth), and iFISH detection. This study was conducted to supplement this, beginning with the clinical symptoms, to detect the role of miR-23/uPA in MM. Our results differentiated patients according to the location and symptoms of MM, and also linked to differences in traditional staging classification. The hyper-methylation of the promoter region of miR-23 can lead to the down-regulation or deletion of uPA expression in MM, which in turn leads to the up-regulation of uPA expression. The results show that the expression trend of uPA in MM cells in blood is similar to that in bone tumor tissues. This indicates that the destruction of bone tissue by MM is a key step in its malignant transformation, and whether it migrates to the blood or forms solid tumor tissue in the bone may be affected by specific conditions represented by the miR-23/uPA axis.

The progress of human malignancies represented by the MM complex is often affected by the ECM or microenvironment (1–6). One sign of malignant transformation of these malignant tumor cells is metastasis and invasion, in which cells destroy the ECM/basal membrane of the primary site through MMPs and achieve invasive growth and migration to other tissues and organs (21). MMPs are vital to the destruction of local tissues, but they are inactive precursors and must be cleaved by uPA to be activated (21). The results of this study show that the expression level of uPA is closely related to the disease progression of MM. The overexpression of uPA in MM cells can promote the invasion of MM cells *in vitro* and *in vivo*. Although uPA-related research is of great importance, there have been very few reports on uPA in MM similar to that shown in this study, and the current publications mainly focus on the interaction between MM cells and intraosseous cells (such as osteoclasts) and the bone marrow microenvironment (22–26). There are no reports on the expression of uPA in MM cells, the effects of uPA on MM cells, or related molecular mechanisms. To explore the possible mechanisms of the high expression of uPA in MM specimens, we found that miR-23 can act on uPA and downregulate the expression level of uPA. The expression trend of miR-23 in MM specimens is opposite to that of uPA. Overexpression of miR-23 in MM cells can inhibit the proliferation and invasion of MM cells by targeting the 3’UTR of uPA. This study reports the function and mechanism of miR-23/uPA in MM cells for the first time. Reports of uPA-related miR mainly focus on miR-193a, mainly the influence of miR-193a on uPA in various malignant tumors (47–51). This research not only expands our understanding of uPA-related miR, but also indicates more options for MM response.

MicroRNA is a type of non-coding RNA molecule transcribed by RNA Pol II (52–54). In mammalian cells, miRNA is an important target for epigenetic research and an important regulator of many important physiological functions, such as cell proliferation, cell differentiation, survival, and cancer (55–57). Additionally, use of miRs is also an important and effective aspect of anti-tumor gene therapy: the full sequence of miRs is chemically synthesized and prepared as lentiviral particles or coated by various liposomes, which can have anti-tumor effects (58, 59). In this way, the lack or deficiency of expression of miRs functioning as tumor suppressors is also among the important mechanisms of cell canceration. DNA methylation is also an important mechanism for the epigenetic regulation of mammalian cells (60–62). Hypermethylation in the promoter region of tumor suppressor genes can lead to the loss of their expression. For example, Ma *et al.* reported that methylation of the miR-34a promoter region in pancreatic cancer cells can lead to the loss of miR-34a expression in pancreatic cancer cells and clinical specimens (21). In this study, the expression levels of uPA and miR-23 were detected, as was the methylation rate of the promoter region of miR-23. Further, a correlation analysis was conducted, which found that the methylation rate of the promoter region of miR-23 is negatively correlated with the expression level of miR-23 and is positively correlated with the expression level of uPA. The data detected in these clinical specimens further confirms the effects of miR-23 on uPA. Nevertheless, we must still elaborate on the causal relationship between the methylation of the miR-23 promoter region and the expression of uPA in the future. Wang et al. found that DNA methyltransferase 1 can mediate the methylation of the miR-338-5p promoter region, cause the loss of miR-338-5p expression, and ultimately cause a high level of ETS-1 in gliomas (63). For this reason, in the future, our research group will investigate (1) DNMT-1 expression detection in MM; (2) the effects of DNMT-1 overexpression or knockdown on the expression of miR-23, and on the miR-23 promoter’s methylation; and (3) the recruitment of DNMT-1 to the CpG islands in the promoter region of miR-23.

In addition, this study featured a novel methodology. First, for the detection of methylation rate, we used the BSP-NGS method to detect the methylation level of the promoter region of miR-23 in MM cells. In the use of this technology, the sequencing chip for second-generation sequencing can be used directly to detect dozens of samples at the same time. In addition, after the BSP step is completed, the methylation site can be sequenced as an SNP-like site, and the accurate methylation rate of any specific site in a specimen can be directly obtained. In addition, for the study of blood tumor cells such as MM, the main research method adopted is to culture cells for proliferation experiments such as MTT or cell counting. Because it is not easy for blood tumor cells to form subcutaneous neutral tissues, it is difficult for researchers to inoculate such cells under the skin of nude mice to form solid tumor tissues for quantitative analyses. In this study, solid tumor tissues were formed by subcutaneously inoculating MM cells of different sources and stages into nude mice. This is consistent with the characteristics of MM itself: as the disease progresses and the degree of malignant transformation increases, MM cells have greater agglomeration and aggregation, eventually forming solid tumors. Only some MM cells with a relatively low degree of malignancy can form solid tumor tissues after inoculation into nude mice. At the same time, the solid tumor tissue that forms is irregular in shape. The transfection of uPA into MM cells or their inoculation with a higher degree of malignant transformation into nude mice can form more uniform tumor tissues, and at the same time, their ability to form tumors is stronger (after inoculation with the same number of cells, the tumor tissues have larger volumes weights). We used image analysis software to identify the shapes of the tumor tissue. Finally, not only did we clarify the functions of uPA in MM, but we also used small-molecule inhibitors of uPA to produce the anti-tumor activity of MM cells. Moreover, the study’s limitations are also acknowledged here: (1) In this study, we found that miR-23 was expressed in MM, while other miRNAs potentially targeting on uPA were expressed at very low or no levels in MM. To this end, we mainly focused on miR-23, while failing to uncover and explore the possible mechanisms for the deficency of expression of other miRNAs in MM; (2) Our results also showed that the methylation of the miR-23 promoter region may be a possible mechanism for the downregulation of miR-23 expression and the upregulation of uPA expression, but we failed to elucidate the possible mechanism of the methylation of the miR-23 promoter region, such as the relationship between DNA methylation transferase (41, 64–66) with miR-23; (3) The promoter methylation of tumor suppressor genes represented by some miRNAs is an important mechanism for the occurrence and progression of malignant tumors (53, 67–69). Small-molecular inhibitors such as DNA methyltransferase can not only upregulate the expression of some tumor suppressor genes, but also interact with the tumor suppressor genes (53, 67–69). Molecular targeted drugs and other anti-tumor drugs are used in combination. This study failed to detect the effect of DNA methyltransferase inhibitors on MM cells, which will be further explored in the future. Despite many advances, the options for MM anti-tumor drug treatment remain limited. It is worth mentioning that in addition to the action of uPA on MMPs, it can also form a complex with uPAP (the uPA-uPAR axis) and invoke non-proteolytic receptor-related pathways participating in the migration/invasion, adhesion, differentiation, tumor progression, or angiogenesis of cancer cells (70). In the future, we will explore the role of uPA/uPAR in MM in depth. Moreover, our future researches will also refer to or include the publicly available data for RNAseq and DNA methylation (71). We also systematically summarize all previous findings from other studies in pubmed that uPA is closely related to the disease progression of MM, and is also an important regulator of the interaction between MM and the tumor microenvironment (22–26, 72–79). This also confirms the results of this study from one side.

## Data Availability Statement

The original contributions presented in the study are included in the article/supplementary material. Further inquiries can be directed to the corresponding authors.

## Ethics Statement

The studies involving human participants were reviewed and approved by Ethics Committee of the General Hospital of Central Theater Command. The patients/participants provided their written informed consent to participate in this study.

## Author Contributions

QR and DW: concept, design, statistics, data collection, manuscript writing, final approval. CH: design, statistics, data collection. DX concept, data collection. QW: statistics, manuscript writing. QR: statistics, data collection. QW and DW: statistics, data collection. DW and QR: concept, design, statistics, data collection, manuscript writing, final approval. All authors contributed to the article and approved the submitted version.

## Conflict of Interest

The authors declare that the research was conducted in the absence of any commercial or financial relationships that could be construed as a potential conflict of interest.

## Publisher’s Note

All claims expressed in this article are solely those of the authors and do not necessarily represent those of their affiliated organizations, or those of the publisher, the editors and the reviewers. Any product that may be evaluated in this article, or claim that may be made by its manufacturer, is not guaranteed or endorsed by the publisher.
